# Advances in Plant–Fungal Pathogen Interaction

**DOI:** 10.3390/plants14111632

**Published:** 2025-05-27

**Authors:** Carlos Garrido, Hernando José Bolívar-Anillo, Victoria E. González-Rodríguez

**Affiliations:** 1 Laboratorio de Microbiología, Departamento de Biomedicina, Biotecnología y Salud Pública, Facultad de Ciencias del Mar y Ambientales, Universidad de Cádiz, 11510 Puerto Real, Cádiz, Spain; 2 Faculty of Basic and Biomedical Sciences, Center for Research on Biodiversity and Climate Change—ADAPTIA, Simon Bolivar University, Barranquilla 080002, Colombia; hernando.bolivar@unisimon.edu.co

## 1. Introduction

The relationship between plants and fungal pathogens is an intricate evolutionary arms race, where dynamic interactions continually shape global ecosystems and agricultural productivity. Plant pathogens, especially fungi, remain among the most critical threats to crop yield, biodiversity, and food security, exacerbated by climate change, globalization, and intensive agriculture. As pathogens evolve rapidly, rendering traditional management strategies less effective, understanding the molecular mechanisms underlying pathogen virulence and plant defense responses becomes increasingly urgent.

In recent decades, plant pathology research has witnessed substantial developments driven by cutting-edge methodologies, including genomics, transcriptomics, proteomics, culturomics, and metabolomics. These multi-omics strategies have fundamentally transformed our capacity to dissect the complex interactions between plants and pathogens. Beyond merely identifying genetic markers and metabolic pathways, these advances have paved the way for developing innovative and sustainable management strategies.

Within this context, our Special Issue, “Advances in Plant–Fungal Pathogen Interaction”, compiles twelve insightful contributions showcasing significant progress in understanding plant–pathogen interactions from molecular, ecological, and applied perspectives.

## 2. Molecular Insights and Omics Approaches

Sirangelo (2024) [1] comprehensively reviewed molecular resistance mechanisms against *Fusarium* head blight (FHB), emphasizing the role of multi-omics technologies. This work highlighted key genetic loci such as *Fhb1* and *Fhb7*, revealing pathways involved in mycotoxin detoxification and cell wall reinforcement, critical for developing resistant wheat cultivars.

Song et al. (2024) [2] examined the molecular mechanisms of rice blast caused by *Magnaporthe oryzae*. Their research revealed how the pathogen effector AvrPik-D directly targets the rice Rubisco Small Subunit (OsRBCS4), effectively suppressing host innate immunity. This discovery provides crucial molecular targets for breeding resistant rice cultivars.

Similarly, da Silva Ripardo-Filho et al. (2023) [3] focused on the fungal pathogen *Botrytis cinerea*, providing a comprehensive review of its secondary metabolism, notably the biosynthesis of sesquiterpenes, diterpenes, and polyketides. These secondary metabolites were confirmed as essential virulence factors, offering valuable targets for developing innovative antifungal treatments.

Shinde et al. (2023) [4] detailed hormonal interactions occurring during Black Knot disease progression in plum, caused by *Apiosporina morbosa*. Their meticulous temporal analysis revealed significant changes in the auxin and cytokinin concentrations, suggesting these hormones as critical biomarkers and potential breeding targets for disease resistance.

## 3. Sustainable Microbial and Biological Control Approaches

Nurzhanova et al. (2024) [5] explored microbial biopreparations based on beneficial bacteria (*Lacticaseibacillus paracasei* M12 and *Bacillus amyloliquefaciens* MB40) against fire blight (*Erwinia amylovora*). Their promising results highlight microbial solutions that stimulate antioxidant systems and enhance photosynthetic activity in fruit trees, thus sustainably boosting disease resistance.

Complementing these findings, Bódalo et al. (2023) [6] explored the biocontrol potentials of endophytic bacteria from ginger (*Zingiber officinale*), specifically against *Botrytis cinerea* and *Colletotrichum acutatum*. They documented significant antimicrobial activity, siderophore production, nitrogen fixation, and plant growth promotion, proposing these bacteria as promising sustainable alternatives to chemical fungicides.

## 4. Pathogen Adaptation, Epidemiology, and Environmental Resilience

Ghoneem et al. (2023) [7] characterized enzymatic and molecular aspects of coriander leaf blight caused by *Alternaria dauci*, emphasizing the pathogen’s aggressive enzymatic arsenal. This work highlights the urgent need for improved resistance and diagnostic methods to mitigate its agricultural impact.

Alors et al. (2023) [8] studied drought-resistant resting cysts produced by *Paraphysoderma sedebokerense*, emphasizing their role in pathogen survival in fluctuating environmental conditions, significantly complicating disease management strategies, particularly under climate change scenarios.

Carezzano et al. (2023) [9] examined bacterial biofilm formation in phytopathogens, highlighting biofilms as essential structures conferring pathogen resilience against stress and complicating traditional disease management approaches, calling for innovative control strategies.

## 5. Mycorrhizal Symbiosis and Beneficial Plant–Microbe Interactions

Gaši et al. (2023) [10] evaluated the beneficial impacts of arbuscular mycorrhizal fungi (*Rhizophagus irregularis*, *Funneliformis mosseae*, and *Funneliformis caledonium*) on grapevine health under viral stress, demonstrating improved photosynthetic performance and enhanced resilience. Their study underscores the potential of mycorrhizal symbiosis as a sustainable biological approach to disease management.

Similarly, Dyshko et al. (2024) [11] presented a detailed overview of mycorrhizal associations in pine trees, emphasizing their critical roles in strengthening plant resilience against biotic and abiotic stresses, advocating the integration of these beneficial fungi in sustainable forestry management.

## 6. Practical Management and Integrated Disease Control

Liu et al. (2023) [12] critically assessed fungicide effectiveness against blackleg disease (*Plenodomus wasabiae*) in wasabi (*Eutrema japonicum*), highlighting discrepancies between laboratory efficacy and practical outcomes in planta. Their findings advocate for integrated disease management combining chemical, biological, and cultural practices to ensure effective long-term control.

## 7. Conclusions and Future Perspectives

Collectively, the contributions of this Special Issue clearly illustrate the diverse and integrative nature of modern plant pathology research. Molecular discoveries, ecological insights, and practical management approaches collectively enrich our knowledge and substantially advance disease control methodologies.

Future research must continue to deepen our understanding of pathogen adaptation mechanisms, exploring innovative and sustainable microbial-based management strategies, including microbial biopreparations and beneficial mycorrhizal associations. Furthermore, the integration of advanced bioinformatics, artificial intelligence, machine learning, and predictive modeling will be crucial in anticipating and effectively managing future disease threats.

Enhanced interdisciplinary collaboration between plant pathologists, agronomists, molecular biologists, ecologists, microbiologists, and bioinformaticians will be essential to translate vital research into practical, sustainable agricultural solutions, ultimately safeguarding global food security and environmental sustainability.

We sincerely thank all contributing authors for their rigorous scientific work, reviewers for their insightful and constructive feedback, and the editorial team of Plants for their diligent support and guidance throughout the editorial process.
